# DnaC Inactivation in *Escherichia coli* K-12 Induces the SOS Response and Expression of Nucleotide Biosynthesis Genes

**DOI:** 10.1371/journal.pone.0002984

**Published:** 2008-08-20

**Authors:** Anders Løbner-Olesen, Monika Slominska-Wojewodzka, Flemming G. Hansen, Martin G. Marinus

**Affiliations:** 1 Department of Science, Systems and Models, Roskilde University, Roskilde, Denmark; 2 Department of Molecular Biology, University of Gdansk, Gdansk, Poland; 3 Center for Biological Sequence Analysis, Department of Systems Biology, Technical University of Denmark, Lyngby, Denmark; 4 Department of Biochemistry and Molecular Pharmacology, University of Massachusetts Medical School, Worcester, Massachusetts, United States of America; University of Massachusetts, United States of America

## Abstract

**Background:**

Initiation of chromosome replication in *E. coli* requires the DnaA and DnaC proteins and conditionally-lethal *dnaA* and *dnaC* mutants are often used to synchronize cell populations.

**Methodology/Principal Findings:**

DNA microarrays were used to measure mRNA steady-state levels in initiation-deficient *dnaA46* and *dnaC2* bacteria at permissive and non-permissive temperatures and their expression profiles were compared to MG1655 wildtype cells. For both mutants there was altered expression of genes involved in nucleotide biosynthesis at the non-permissive temperature. Transcription of the *dnaA* and *dnaC* genes was increased at the non-permissive temperature in the respective mutant strains indicating auto-regulation of both genes. Induction of the SOS regulon was observed in *dnaC2* cells at 38°C and 42°C. Flow cytometric analysis revealed that *dnaC2* mutant cells at non-permissive temperature had completed the early stages of chromosome replication initiation.

**Conclusion/Significance:**

We suggest that in *dnaC2* cells the SOS response is triggered by persistent open-complex formation at *oriC* and/or by arrested forks that require DnaC for replication restart.

## Introduction

The initiation of chromosome replication is a highly regulated process that occurs once per cell cycle at the origin of replication, *oriC*, and involves many proteins including DnaA and DnaC. The DnaA protein, with either ADP or ATP, initiates the process by binding to its 9-mer recognition sequences within *oriC* [1], which results in opening of the AT rich region. The open complex is stabilized by binding of DnaA-ATP to the single stranded regions [2]. The bound DnaA protein recruits the hexameric DnaB helicase associated with ATP bound DnaC to the single stranded region. Subsequently, DnaC loads the DnaB helicase on the open complex to promote further duplex opening [3]. In this process ATP is hydrolyzed and DnaC is released. Finally two or three polymerase III holoenzyme molecules are loaded at the origin to duplicate the chromosome [4].

During the elongation phase of chromosome replication, replication forks can stall at sites of spontaneous or induced damage and repair of such forks induces the SOS response [5]. The primosomal proteins, PriABC and DnaT, are required to load DnaC at the reconstructed fork [6]. In *dnaC2* bacteria at the non-permissive temperature, about 18% of forks are unable to terminate [7].

The DnaA protein can also act as a transcriptional activator/repressor of several genes including *dnaA*, *mioC*, *rpoH* (heat shock sigma factor), *uvrB*, *proS*, *nrd* (nucleotide diphosphate reductase), *glpD* and *fliC* by binding to 9-mer sequences in the promoter regions (reviewed in [8]). The *nrdAB* genes are also subjected to cell cycle control through a 45 bp inverted repeat, located upstream of the DnaA 9-mers, and which may be the target of the YbaD (NrdR) protein [9]–[11] . Other cell cycle controlled genes have been reported including: *dam* (DNA adenine methyltransferase), *mukB* (chromosome condensation), *seqA* (origin sequestration), *ftsQ* and *ftsZ* (cell division) [12], [13] as well as *gid* and *mioC* [14] and *hns* [15]. The mechanism by which cell cycle regulation occurs at these gene loci, however, is not known.

Bacterial cell cycle control of genes is best understood in *Caulobacter crescentus*, an organism which has defined morphological cell forms. Three transcriptional regulatory proteins, DnaA, GcrA and CtrA, acting sequentially regulate more than 200 cell cycle-regulated genes [16]. This is achieved by the sequential location of these regulator genes on the chromosome and through the use of DNA methylation to control gene expression; for example, the *dnaA* promoter is expressed only when DNA is fully methylated while hemimethylation promotes transcription of *ctrA* [17].

In addition to cell cycle control, there appears to be a correlation between initiation of chromosome replication and the expression of the *nrdAB* genes (reviewed in [18]). Gon et al [19] found that suppressor mutations in a thioredoxin-glutaredoxin mutant mapped to an operon encoding *dnaA* and *dnaN*. These and other data led to a model in which DnaA-ATP negatively regulates *nrdAB* expression while DnaA-ADP is without regulatory effect. A high DnaA-ATP level is expected at the time of initiation but this should decrease as elongation commences through the action of the Hda protein which converts DnaA-ATP to DnaA-ADP [20]. The decrease in DnaA-ATP should result in increased transcription of *nrdAB*. The interplay of DnaA-ATP, Fis, IciA and NrdR on transcription of the *nrdAB* genes, however, occurs in a manner that is incompletely understood [18].

Incubation of temperature-sensitive strains, such as *dnaC* and *dnaA*, under non-permissive conditions is expected to induce expression of heat shock genes which are controlled by an alternative sigma factor, RpoH [21]. As noted above, the expression of the *rpoH* gene can also be influenced by DnaA. Some heat shock response genes overlap with the SOS response which is induced by DNA damage through the persistence of single-stranded DNA which allows for the formation of RecA filaments and activation of its co-protease activity resulting in cleavage of the LexA repressor and activation of SOS gene transcription [22].

In this communication, we explore the events that occur in a *dnaC2* (Ts) strain incubated at the non-permissive temperature. We find that there is induction of the heat shock and SOS responses and of genes involved with nucleotide biosynthesis and utilization as well as the *dnaC* gene itself. In the control *dnaA46* (Ts) mutant, there is also induction of the heat shock response and nucleotide biosynthesis and utilization genes as well as the *dnaA* gene itself but not the SOS response. We were not able to identify cell cycle regulated genes in *dnaC2* synchronized cells using a gene expression microarray approach.

## Materials and Methods

### Bacterial strains

The strains used were MG1655 [23], MG1655 *dnaC2* [24] and MG1655 *dnaA46 tna*::Tn*10*. The latter strain was constructed for this work by P1 transduction.

### Cell culturing

Strain MG1655 and its *dnaA46* and *dnaC2* derivatives were grown exponentially at permissive (30°C) temperature in AB minimal medium [25] supplemented with 1 µg/ml thiamine, 0.2% glucose and 0.5% casamino acids. At an optical density of about OD_450_ = 0.3, the cultures were shifted to 38°C and 42° for *dnaC2* and to 42° for *dnaA46* and both temperatures for MG1655 for 90 minutes before harvesting and RNA extraction. During this period ongoing chromosomal replication was completed in the *dna* mutants and the majority of cells divided so that most cells contained one genome equivalent. The cultures were diluted regularly so that the OD_450_ never exceeded 0.4.

In order to identify cell cycle regulated transcripts, *dnaC2* cells were cultured as above and a single round of initiation was allowed to occur by shifting the culture at t = 0 to 30°C for 10 minutes, before continued incubation at 38°C. Samples were taken for RNA isolation at times t = −1, 10, 20, 30, 40, 50, 60 and 80 minutes.

To determine if initiation of chromosome replication had occurred in the *dnaC2* strain during incubation at the non-permissive temperature, cells were incubated for 90 minutes at 38°C and 300 µg/ml rifampicin and 10 µg/ml cephalexin added to the culture to prevent further initiation and cell division, respectively. Incubation was continued for 4 hours at either 30°C or 38°C prior to flow cytometric analysis [26].

### RNA isolation and processing

The procedure for mRNA isolation examining cell cycle gene expression was that recommended for the Affymetrix GeneChip *E. coli* Genome 1.0 array. Culture samples were removed at t = −1, 10, 20, 30, 40, 50, 60 and 80 minutes as described above and total RNA isolated using RNeasy Total RNA Isolation Kit (Qiagen). rRNA was removed from 100 µg total RNA by annealing rRNA specific primers, extending with MMLV Reverse Transcriptase (Epicentre Technologies) and digesting the RNA-DNA hybrids with RNAse H (Epicentre Technologies). The enriched mRNA was fragmented by heat and end-labeled with Biotin using T4 Polynucleotide Kinase (New England Biolabs). The end-labeled RNA was hybridized to *E. coli* K-12 GeneChip 1.0 arrays in a GeneChip Fluidics Station (Affymetrix). The arrays were scanned using an HP Gene Array Scanner. The t = −1 culture sample was used as the baseline for all others in the series. Full details of this procedure are available at the Affymetrix website (www.affymetrix.com).

The procedure for all other experiments was that recommended for the Affymetrix GeneChip *E. coli* Antisense Genome Array. Total RNA was isolated using the MasterPure RNA Purification kit (Epicentre Technologies). Random primers (Invitrogen) were annealed to 10 µg of the RNA and SuperScript II Reverse Transcriptase used for cDNA synthesis. Residual RNA was removed by alkaline hydrolysis and the cDNA recovered by isopropanol precipitation. The cDNA was fragmented with DNAse I (Amersham Biosciences) in One-Phor-All buffer (Amersham Biosciences) and end-labeled with Biotin using the Enzo BioArray Terminal Labeling Kit. The end-labeled RNA was hybridized to Affymetrix GeneChip *E. coli* Antisense Genome arrays in a GeneChip Fluidics Station (Affymetrix) and scanned in a Affymetrix GeneChip Scanner 3000. Full details of this procedure are available at the Affymetrix website (www.affymetrix.com).

### Data collection and analysis

The fluorescent labeled RNA or cDNA from the *E. coli* MG1655 strains was scanned exactly as described in the Affymetrix User Guide (www.affymetrix.com) and analyzed using GeneChip Analysis Suite software. Raw data were exported as text files and imported into Microsoft Excel (Office 2003) in which further sorting was accomplished. In the comparison of 4,242 gene encoding proteins in the *dnaA* and *dnaC* strains, we required that all genes should have a “present call”. Furthermore, we required that the actual numbers compared in the analysis should be above a (more or less arbitrarily chosen) threshold. For the comparison analyses shown in the Figures 2, 3, 5, 6 of this article we chose 200 scanning units as the minimal threshold. Using this threshold value, we typically obtained present calls for between 3200 to 4000 genes. The average expression for a given probe set (including those with an absence call or negative value) was 385 scanning units and the highest expressed genes (encoding ribosomal proteins and other components of the protein synthesis apparatus) were around 5000 scanning units. The results are from duplicate trials. All data from the microarray analysis can be found at http://users.umassmed.edu/martin.marinus/arrays/.

## Results

### Gene expression in synchronized cultures

To identify genes that are specifically expressed at certain times of the cell cycle, we analyzed global gene expression in synchronized cells. If such genes exist they could putatively be responsible for proper cell cycle progression.

**Figure 1:**
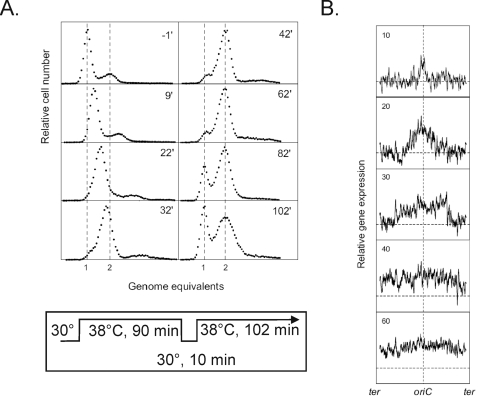
Global transcription profiles and DNA content. A culture of *dnaC2* cells growing at 30°C was shifted to 38° for 90 min, 30°C for 10 min, and returned to 38°C for 102 min. At the indicated times samples were removed for flow cytometry (40,000–50,000 cells) (A) and transcription profiling using microarrays (B). In panel B, a moving average of the expression of fifty genes is located as on the genetic map with *oriC* at the center.

*E. coli* MG1655 cells were synchronized with respect to chromosome replication initiation using the temperature sensitive *dnaC2* allele. When *dnaC2* cells were shifted to non-permissive temperature (38°C) for 90 minutes, initiations ceased whereas cell division and mass increase continued. Consequently most cells contained one fully replicated chromosome (Figure 1A) where t = −1 is one minute before the downshift to 30°C. At time t = 0, cells were shifted back to permissive temperature (30°C) for 10 minutes to ensure a single round of synchronous initiation, after which the temperature was increased to 38°C to prevent multiple initiations. Flow cytometric analysis confirmed that a single round of initiation had taken place in each cell because the cellular DNA content of virtually all cells increased from one to two genome equivalents over a period of about 40–50 minutes (Figure 1A). Cell division followed some time after completion of replication since cells containing one genome equivalent re-appeared at t = 82 minutes.

Global gene expression was monitored in samples taken at various times before and after initiation of replication in the synchronous culture. RNA was extracted from each sample, hybridized to Affymetrix GeneChip *E. coli* Genome 1.0 arrays and all data was normalized to the expression level of cells that had not initiated replication (t = −1). In Figure 1B, relative gene expression is plotted against location of genes on the chromosome where the origin is at the center of the ordinate. There is an initial bidirectional increase in expression of genes at the origin which continues around the chromosome and is completed in about 40 min. These data correlate well with the increase in DNA content shown in Figure 1A where chromosome duplication is complete in about 40 min and an increase in cells with a single genome equivalent is apparent at 82 min as a consequence of cell division.

An examination of the data in Figure 1B did not yield significant cell cycle related changes in expression of any individual gene over the course of the experiment. The *dam*, *nrdAB*, *dnaA*, *mioC*, *gidA*, *ftsQ*, *ftsZ* and *hns* genes have previously been reported to be expressed in a cell cycle dependent manner [12]–[15], [27] but the microarray analysis failed to confirm this, perhaps because not enough data points were taken throughout the experiment.

We observed, however, that there were changes in global gene expression of the sample taken at non-permissive temperature (t = −1) relative to a sample from exponentially growing cells at the permissive temperature. These included the heat shock and SOS regulons, nucleotide biosynthesis genes and the *dnaC* gene itself. These are described in detail below.

### Induction of heat shock genes

**Figure 2:**
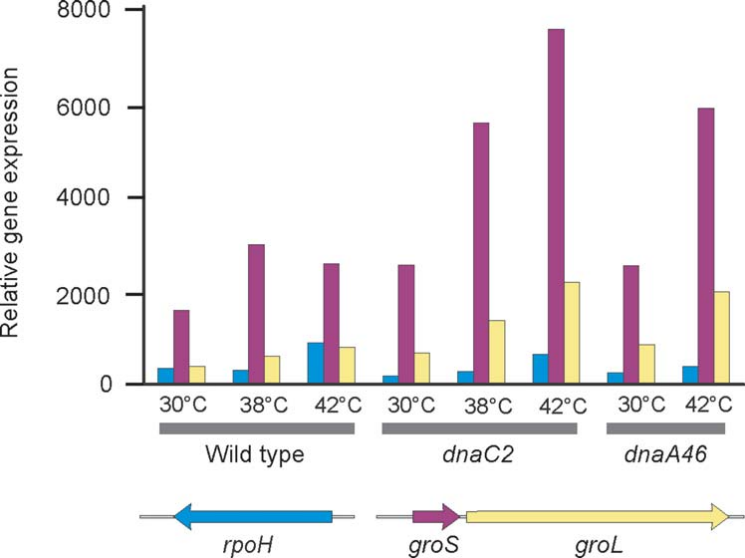
Relative gene expression of heat shock genes in the wildtype, *dnaA46* and *dnaC2* strains. Bacteria were grown at 30°C, 38°C or 42°C for 90 min, harvested and total RNA extracted for microarray analysis. The *groS* and *groL* genes, but not *rpoH*, are part of the heat-shock regulon.

As an internal control for the validity of the analysis we examined expression of selected genes belonging to the heat shock regulon in samples of *dnaC2* cells taken at 30°C and after 90 minutes at 38°C. In this experiment we included samples of wild-type cells and *dnaA46* cells taken at 30°C and after 90 minutes at 42°C. Growth at elevated temperature should induce the heat shock response and we found that expression of *rpoH*, encoding the heat shock response sigma32, and *groES* and *groEL* chaperone genes (among others such as *hfq*) was increased as shown in Figure 2. Expression of *rpoH* in *dnaC2* cells was increased about 3-fold in cultures grown at 38°C compared with cells grown at 30°C and the corresponding changes were about 2.5-fold and 2-fold for the wild-type and *dnaA* cells (Figure 2). Increased signal on the array of the *groES* and *groEL* genes showed changes of a similar magnitude in the *dnaC2,* wild-type and *dnaA46* strains (Figure 2). We conclude that these data indicate that modest changes in steady-state mRNA levels were readily detected by this method of analysis.

### Microarray results

For wildtype cells, 297 genes showed greater than 2-fold increased steady-state mRNA levels at either 38°C or 42°C. In addition to the heat shock genes mentioned above these included genes encoding proteins for stress responses (e.g., *uspA*, *D*, *E*; *cspABEG*, *C*, *D*, *F*, *I*), chromosomal architecture (e.g., *hns*, *himAD*, *hupAB*), central metabolic pathways (e.g., *talB*, *thr*, *trp*, *trx*) and cell membrane (*lpp*, *pal*, *bolA*) (see Table S1 for complete listing).

The corresponding numbers for *dnaA46* and *dnaC2* cells were 227 and 140 (see Table S1 for complete listing). Loss of DnaC activity resulted in induction of genes involved in nucleotide metabolism, SOS response, *dnaC* itself (see below) as well as genes in central metabolic pathways. Inactivation of DnaA function led to de-repression of genes negatively regulated by DnaA including *dnaA* itself, *mioC*, *rpoH*, *uvrB*, *proS*, *nrd*, *glpD* and *fliC* as observed previously [8], as well as genes involved in nucleotide metabolism (see below and Table S1 for complete listing). Among the genes showing the greatest increase in mRNA abundance at the non-permissive temperature in *dnaA46* cells were those encoding enzymes involved in fatty acid and phospholipid metabolism. Genes *plsX-fabH-fabD-fabG* (*pls* = phospholipid synthesis, *fab* = fatty acid biosynthesis) form a transcriptional unit which may also include the downstream co-transcribed *fabF* and *pabC* genes which form a transcriptional unit under a separate promoter. Genes *plsX*, *fabH*, *fabD*, *fabF* and *pabC* showed 9.9, 7.0, 5.1, 4.5 and 3.2- fold increases respectively in *dnaA46* cells incubated at 42°C relative to 30°C. The unlinked genes *fadL* and *fabB* showed 9.9 and 5.0-fold increases (*fad* = fatty acid degradation). The increased mRNA abundance for the *pls*, *fab* and *fad* genes was not detected in wildtype and the response was much reduced in the *dnaC2* strain (6.6 for *fadL* and 3.3 for *fadB*).

### Loss of DnaC activity leads to SOS induction

**Figure 3:**
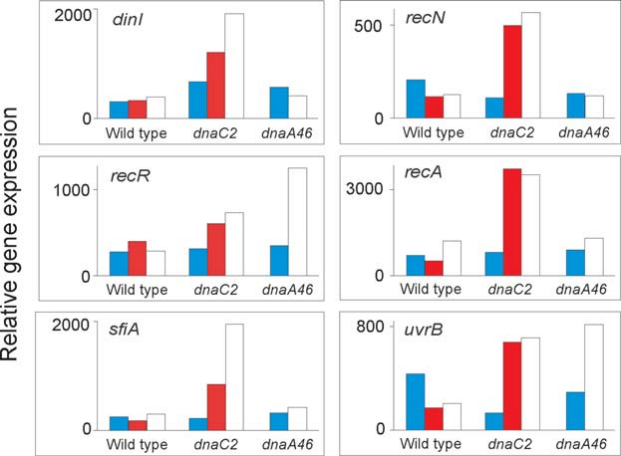
SOS induction in *dnaC2* bacteria at the non-permissive temperature. Wildtype, *dnaA46* and *dnaC2* bacteria were grown at 30°C (blue), 38°C (red) or 42°C (white) for 90 min, harvested and total RNA extracted for microarray analysis. SOS genes with at least a 2-fold increase in steady-state mRNA levels in the *dnaC2* strain at the non-permissive temperature are shown. The plots are normalized to the sample giving the highest scanning signal.

A number of genes had increased expression in the *dnaC2* mutant at non-permissive temperature. One distinct group of these belonged to the SOS regulon [22]. SOS genes that show increased transcription in *dnaC2* cells include *recA*, *recN*, *sulA* and *uvrB* (Figure 3). Although two genes (*recR* and *uvrB*) also showed increased transcription at 42°C compared to 30°C in the *dnaA46* mutant, there was no coordinated response of the SOS regulon in this strain. No SOS induction was observed in wild-type cells at 42°C. Therefore the SOS induction observed is specific to the *dnaC2* strain and did not result from incubation at 42°C nor to cessation of DNA synthesis at termination sequences.

### Open complex formation occurs in *dnaC2* cells

**Figure 4:**
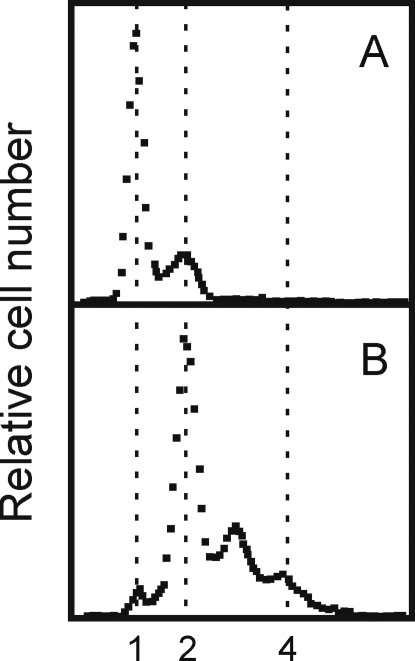
Origins are active in a *dnaC2* mutant after 90 min incubation at 38°C. The DNA distributions in a *dnaC2* culture incubated at 38°C for 90 min after synchronous initiation (A) followed by addition of rifampicin and cephalexin and growth at 30°C for 3 hr (B).

To determine if initiation had occurred in *dnaC2* cells at non-permissive temperature, these were incubated for 90 minutes at 38°C before addition of rifampicin and cephalexin to prevent further initiation and cell division, respectively. Incubation was continued for 3 hours at either 30°C or 38°C prior to flow cytometric analysis. Cells kept at non-permissive temperature contained mostly one fully replicated chromosome and some cells with two (Figure 4A). When replication was allowed to proceed to completion at permissive temperature, most cells contained two fully replicated chromosomes and some had three and four chromosomes but very few had only one (Figure 4B). Therefore the *dnaC2* cells maintained at non-permissive temperature had been arrested at a late stage of initiation process where DnaA protein synthesis was no longer required, i.e., initiation had taken place, and replication could continue in the presence of rifampicin when DnaC was re-activated at permissive temperature. These results confirm previous demonstrations of open complex formation in *dnaC2* cells using different techniques [28], [29].

Because *dnaC2* mutant cells are deficient in loading the DnaB helicase at the origin, these are likely to be arrested at the open complex stage of the initiation process, where single stranded DNA is present. The single-stranded DNA of the open complex is probably a signal for SOS induction in the absence of functional DnaC protein. Loading of DnaC by primosomal proteins occurs at arrested replication forks [5] and in the *dnaC2* mutant Maisnier-Patin et al [7] showed that if active DnaC is not available during elongation about 18% of the cells fail to complete replication. This is the probable explanation for the incomplete separation of peaks showing 2–3 and 3–4 genome equivalents in Figure 4B indicating that in some cells replication may have stopped before forks reached the termini. The formation of such inactivated forks can lead to SOS induction [5]. The induction of the SOS response we have observed in *dnaC2* bacteria likely derives from arrested forks and/or from persistent open complex formation at *oriC*.

### Induction of the *dnaT* and *dnaC* containing operon

**Figure 5:**
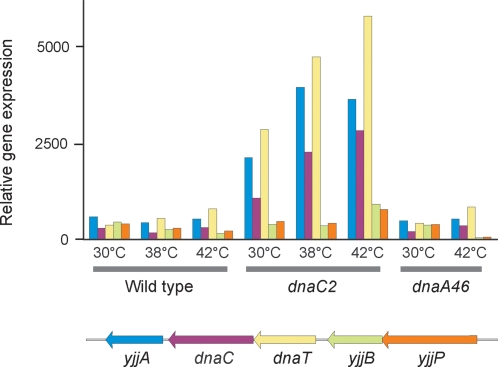
Transcription of the *dnaT* operon in wildtype, *dnaA46* and *dnaC2* strains. Wildtype, *dnaA46* and *dnaC2* bacteria were grown at 30°C, 38°C or 42°C for 90 min, harvested and total RNA extracted for microarray analysis. The *yjjB* and *yjjP* genes are not part of the *dnaT* operon.

The *dnaC* gene is in an operon consisting of the *dnaT*, *dnaC* and *yjjA* genes downstream from the *yjjB* and *yjjP* genes which are not in the same transcriptional unit (Figure 5). In the *dnaC2* mutant, there is a high basal level of transcription from the *dnaC* operon even at 30°C and this is increased 2–2.5 fold at the higher temperatures (Figure 5). The increased expression was not the result of SOS induction because addition of nalidixic acid, a known SOS inducer, did not lead to increased transcription [30]. In the wildtype and *dnaA* mutant, there was no difference in the steady-state level of transcripts from the *dnaC* operon at high and low temperatures.

This result indicates that the *dnaC* operon is regulated by neither the SOS response nor temperature, but seems to be autoregulated although the precise mechanism is unknown. In the *dnaA46* mutant, we found increased expression of the *dnaA* operon at the high temperature (data not shown) confirming autoregulation of this gene [31].

### Nucleotide biosynthesis genes

**Figure 6:**
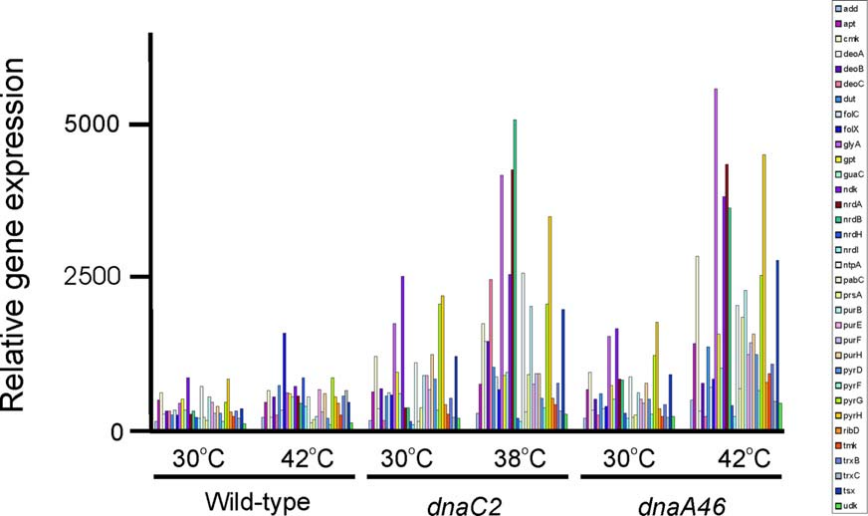
Increased mRNA steady-state levels for nucleotide biosynthesis genes. Wildtype, *dnaA46* and *dnaC2* strains were incubated for 90 min at 30°C, 38°C or 42°C before harvesting and total RNA extraction for microarray analysis.

We observed that a number of genes involved in nucleotide biosynthesis had at least a 2-fold increased level of steady-state expression in the *dnaC2* mutant at non-permissive temperature (Figure 6). The same genes also showed increased expression in the *dnaA46* mutant at non-permissive temperature, but not in wild-type cells at 42°C (Figure 6). High values of expression were obtained for *nrdA* (5.2, 11.8) and *nrdB* (4.4, 13.9) encoding ribonucleotide diphosphate reductase in *dnaA* and *dnaC* strains respectively. The spectrum of genes with increased transcription covered all aspects of nucleotide biosynthesis and interconversion (Figure 6).

Because this group of genes was de-repressed in both *dnaC2* and *dnaA46* mutant cells at non-permissive temperature, it is conceivable that the signal for this induction is generated by the lack of DNA elongation. We aligned the known promoter regions of derepressed genes with respect to the +1 position and searched for common motifs using the predictive transcription binding site program of McCue et al [32] but none were found (Table S1).

## Discussion

We used DNA microarrays to measure gene expression in cells where DNA synthesis was arrested. We have interpreted the increased signal from the arrays for particular genes as evidence of increased transcription initiation rather than increased message stability. Although the latter may be an important factor, it is unlikely as an explanation for all the genes that were monitored. Temperature-sensitive mutations in the *dnaA* or *dnaC* genes were used to stop the initiation process at different stages, i.e., before loading DnaB helicase. Incubation of both *dnaA46* and *dnaC2* mutants at the non-permissive temperature led to a substantial increase in the steady-state mRNA levels for genes involved in nucleotide biosynthesis and nucleotide interconversion. At the time of sampling at the non-permissive temperature, however, cells were no longer replicating their DNA (Figure 1A) although the signal for gene de-repression could have been generated during chromosome replication. The results suggest an unknown regulatory response directly linking chromosome replication and nucleotide biosynthesis where cessation of replication leads to increased expression of nucleotide biosynthetic genes. Although counter-intuitive, the response is reminiscent of early studies on regulation of amino acid biosynthesis where starvation for a particular amino acid leads to derepression of the genes in the biosynthetic pathway for that amino acid [33].

Further evidence suggesting a link between chromosome replication and expression of nucleotide biosynthesis genes was obtained from microarray data measuring the induction of genes in the SOS regulon after exposure of cells to UV-irradiation [34]. However, the increased signals from the nucleotide biosynthetic genes was greater in a *lexA*(Ind) mutant than in wildtype indicating that the increases were independent of the LexA regulon. This result confirmed a previous study showing that the specific activity of ribonucleotide reductase was increased in *dnaB* and *dnaE* mutants at the non-permissive temperature (a condition that induces the SOS system in these mutants) or in wildtype cells challenged with either nalidixic acid or bleomycin [35]. The increase in ribonucleotide reductase activity, however, was not affected by mutations in the *recA* or *lexA* genes [35]. Another study using *nrd*::*lac* fusions on multicopy plasmids and hydroxyurea exposure in wildtype indicated a substantial induction of beta-galactosidase after treatment which was substantially LexA-independent [36]. Taken together, all the studies are compatible with a model in which replication fork arrest leads to SOS-independent increased expression of nucleotide biosynthesis genes. The mechanism by which this occurs is unknown.

Replication fork arrest is an explanation for induction of SOS and perhaps for nucleotide biosynthesis genes in *dnaC2* mutants. However, the increased expression of the same nucleotide biosynthesis genes in the *dnaA46* mutant indicates that another mechanism must exist. One possibility is that expression of these genes is normally repressed by DnaA. Apart from the *nrd* genes, we know of no data to support such a regulatory scheme and there are no DnaA boxes in the promoters of nucleotide biosynthesis genes listed in Table S1. Alternatively, this other mechanism is common to both mutant strains incubated at the non-permissive temperature for 90 min where one round of chromosome replication and cell division occur (Figure 1) and the cells are poised to re-initiate replication. We suggest that under these stressed conditions some mechanism coordinating nucleotide biosynthesis and chromosome replication is inactive. It may be related to that operating when DNA elongation is interrupted by spontaneous or induced replication-blocking lesions.

The increased steady-state mRNA levels of nucleotide biosynthetic genes observed in *E. coli dnaA46* and *dnaC2* bacteria at the non-permissive temperature appears to be at variance with a similar study with an initiation-defective *dnaD23*(Ts) strain of *Bacillus subtilis* [37]. DnaD, which has no counterpart in *E. coli*, is part of the PriA primosome [38], interacts with DnaA [39] and has DNA remodeling activity [40]. After 90 min incubation of *B. subtilis dnaD23* cells at the non-permissive temperature, no change in activity of nucleotide biosynthetic genes was observed [37] except for the *nrdEF* genes which showed 2.7 and 3.1-fold increases. Furthermore, mRNA levels for *dnaA* and *dnaN* (beta sliding clamp) were substantially decreased (7.4 and 5.5-fold respectively). This contrasts with the findings described here for the *E. coli dnaA46* strain in which mRNA levels for both *dnaA* and *dnaN* showed increases (3.6 and 3.4-fold respectively). We have no satisfying explanation to reconcile these differences in results obtained with *B. subtilis* and *E. coli*.

Although both *dnaA46* and *dnaC2* cells were arrested at early stages of the initiation process, there were differences in their gene expression profiles. The first finding from our study was the induction of the SOS response in *dnaC2* cells at the non-permissive temperature. The established requirement for DnaC in PriABC-dependent replication restart at arrested forks and its associated SOS induction during fork re-construction [5] argue that this is a source of the inducing signal although only about 18% of forks fail to terminate in *dnaC2* cells at non-permissive temperature [7]. The lack of the SOS response in the wildtype and the *dnaA46* mutant coupled with the demonstration (Figure 4) that the DnaA step in the initiation process had been completed, suggests that a persistent open complex at *oriC* can also be an inducing signal. The open complex is present in every cell and is expected to contain single-stranded DNA, that serves as the inducer for the SOS response by activating the co-protease activity of RecA [22]. Evidence for persistent single-stranded DNA at *oriC* in *dnaC2* cells incubated for 60 min at 40° C was demonstrated by Gille and Messer [28] who showed that KMnO_4_ hypersensitive sites were detectable in the right *oriC* 13-mer of a low copy *oriC* plasmid. The hypersensitivity disappeared after return of the cells to the permissive temperature.

A previous study using an unusual *dnaC* mutant (*dnaC1331*), which is not conditionally-lethal for growth, indicated a three-fold increased level of SOS induction as measured by using a *sulA*::*lac* fusion {Harinarayanan, 2004 1222 /id}. This induction was suggested to be due to inability of DnaC1331 to be loaded by the PriA-PriB pathway for replication restart. A subsequent and more detailed analysis of this mutant strain, however, failed to confirm the increased SOS induction {Boonsombat, 2006 1223 /id}.

We also found an enhanced steady-state mRNA level of the *dnaT*-*dnaC-yiiA* operon in the *dnaC2* strain growing at both 30°C and 38°C (Figure 5). This result suggests that like *dnaA*, the *dnaC* gene is autoregulated. We know of no studies at the *dnaT* or *yjjB* operon promoter regions bearing on this hypothesis.

The up-regulation of genes containing DnaA boxes in their promoter regions was detected only in *dnaA46* bacteria at the non-permissive temperature. These included *dnaA*, *mioC* and *uvrD*, confirming earlier results obtained by different experimental approaches [1]. The increased level of *nrdAB* mRNA in the *dnaA46* strain supports the hypothesis that DnaA acts as repressor of this operon [19] and not as a transcriptional activator [27]. The observation of increased transcription of fatty acid synthesis and breakdown genes in the *dnaA46* strains relative to *dnaC2* and wildtype was unexpected. Although membrane cadiolipin has been reported to activate DnaA [41], it is unclear if this observation is related to the altered transcription of fatty acid genes described here. Alternatively, both *dnaA* [42] and *fabHDG* [43] gene transcription are subject to *relA* (ppGpp synthesis) control and the increased activity of these genes in the *dnaA46* strain at 42°C may be related to it.

When *dnaC2* cells that had been arrested at initiation were returned to the permissive temperature, all cells initiated chromosome replication in synchrony. In these synchronized cells, we were not able to detect cell cycle regulated genes using microarrays. This result is in contrast to previous publications showing cell cycle dependence for a variety of genes (*nrdAB*, *dam*, *mukB*, *seqA*, *ftsQ*, *ftsZ*, *gid*, *mioC* and *hns*) [12]–[15]. For some of these genes (*gid*, *mioC*, *ftsQ*, *ftsZ*) this may be a reflection of gene sequestration by SeqA [44] which is expected to reduce transcription for about one third of the cell cycle [45]. For others (*nrdAB*), the reduced level of Dna-ATP after initiation should increase expression of these genes but as the level of Dna-ATP increases *nrdAB* transcription should decrease. In general, these previous studies showing cell cycle dependence used similar synchronization methods to that used here and transcripts were detected by S1 nuclease protection of specific probes. It is possible that the array method is not as sensitive to detect the approximately 2- to 5-fold differences found in previous studies. Another possible explanation for our failure to detect these and other genes may be that our sampling interval (10 min) was too long thereby missing the window during which the alteration of steady-state mRNA levels occurred. Alternatively, it is unclear what advantage cell cycle regulated genes confer on an organism such as *E. coli* with a short doubling time and generally stable proteins. For the cell cycle regulated genes mentioned above, the consequences of constitutive expression are unknown. However, a 10-fold increase in the level of Dam methyltransferase produced from a multicopy plasmid, which is expected to abrogate the effects of cell cycle dependence, has no obvious deleterious effect on cell growth [46], [47].

In *Caulobacter crescentus*, where the cell cycle is longer and tied to defined morphological changes, master transcriptional regulators control cell cycle-dependent gene expression [16], [17]. DNA methylation by the CcrM methyltransferase is an essential feature of cell cycle regulation. If a similar mechanism operates in *E. coli*, our method would not have detected it. It might be expected, however, that in a *dam* mutant where no hemi- or full methylation is possible such cell cycle regulation might be abrogated if transcriptional regulators can bind only to specific states of methylated DNA. We know of no data indicating an altered cell cycle in *dam* mutants.

## Supporting Information

Table S1 (0.03 MB DOC)
