# Supplementary material for: DnaC Inactivation in Escherichia coli K-12 Induces the SOS Response and Expression of Nucleotide Biosynthesis Genes
Source: PLoS One. 2008 Aug 20;3(8):e2984. doi: 10.1371/journal.pone.0002984 (PMC2500167; doi:10.1371/journal.pone.0002984)
Supplement: Table S1 — (0.03 MB DOC) [file pone.0002984.s001.doc]

Table 2. Promoter alignments. We aligned the known promoter regions of derepressed nucleotide biosynthetic genes with respect to the +1 position and searched for common motifs using the predictive transcription binding site program of McCue,L.A., Thompson,W., Carmack,C.S. and Lawrence,C.E. (2002) Factors influencing the identification of transcription factor binding sites by cross-species comparison. *Genome Res.*, **12**, 1523-1532.

Gene Promoter sequence Regulator protein

+1

cmk CGCTATAATTGCGCAATAAATCCCCATCTGAATACAGACAAAACTGGTTTTTGCACACAACGTTAACGATTTGTGGCGTCGGCGCGTATAATGCGCGCGG T

deoCp1 CGATATTGGCGTGCATAAAGGCGTCTGGCAGGGTTCTGTCGAGGTAACGCCAGAAACGTTTTATTCGAACATCGATCTCGTCTTGTGTTAGAATTCTAAC A DeoR

deoCp2 TTGAACCAGATCGCATTACAGTGATGCAAACTTGTAAGTAGATTTCCTTAATTGTGATGTGTATCGAAGTGTGTTGCGGAGTAGATGTTAGAATACTAAC A CRP-cAMP, CytR, DeoR, Fis, ModE-MoO4

deoCp3 GGCGAAAGCGGTGAAAGCGGCAATTAAACTTGCCGATAAAGCACCGGAAAGCACACCAACTGTCTATCGCCGTATCAGCGAATAACGGTATACTGATCTG A

dut AACGTATCCGTAAAAACCTTGATCTGATCTGCGCGAACGATGTTTCCCAGCCAACTCAAGGATTTAACAGCGACAACAACGCATTACACCTTTTCTGGCA G

glyA GCTCTTTATTCTCCAAAGCCTTGCGTAGCCTGAAGGTAATCGTTTGCGTAAATTCCTTTGTCAAGACCTGTTATCGCACAATGATTCGGTTATACTGTTC G MetR, PurR

nrdA CACCTTGTCTGACCTAAGGTGCGCGAAAGCCACTTTTTCCTTCCTGAGTTATCCACAAAGTTATGCACTTGCAAGAGGGTCATTTTCACACTATCTTGCA G ArgP, CRP-cAMP, Dna-ATP, Fis

nrdH TCCCCCTACCCCGTCACGCTCATATCCAGGGTAATTTCGACCACTATTTGCTATATATTGTGTGGTTGAATCTTTTTTCAACTACATCTAGTATCTCTGT A Fur

nudB TGTGAAGAAGGCTGAGAATAACTGATATGACTCAAATACACGAAATCATTCGCGTTGCATCGAGGCGGCAACTGAGTGAACTCCCATGAGCATAGATAAC T

(ntpA)

prs ACGTTGCTAAAATGTGAATTCAGCAATGATTGCGAGGTTATCGCAAGAAAACGTTTTCGCGAGGTTGATGCGGTGCTTTCCTGGCTGTTAGAATACGCCC C

purB GAAGTGATTTTCGATGAACCGGTTGCCGCCGTGACGCCGGGCCAGTCTGCCGTCTTCTATAACGGTGAAGTGTGCCTCGGTGGCGGTATTATTGAGCAGC G PurR

(hflD)

purE TGAGCTGATTCATTTTCCGTTTTAAAAAACCCGCAACTTTGCTGATTTCACAGCCACGCAACCGTTTTCCTTGCTCTCTTTCCGTGCTATTCTCTGTGCC C PurR

purF AAAAGTCGATGGCGTTGAATATTTTTTCAGCGCCATTTTTATTGATGCGCGGGAAGGAAATCCCTACGCAAACGTTTTCTTTTTCTGTTAGAATGCGCCC C PurR

(cvp)

purH TTCTGTATTTTGCCCACGGTAACCACAGTCAAAATTGTGATCACCATTGAAAGAGAAAAATTCGCGAGCGTTGCGCAAACGTTTTCGTTACAATGCGGGC G PurR, PhoP

pyrD CAAAGAAGGAGCAAAATCTGCCCTGAAACAGGTTCGGAAAACGTTTGCGTTTTTTTTGCCGCAGGTCAATTCCCTTTTGGTCCGAACTCGCACATAATAC G PurR

(multiple starts)

pyrF TAATGTTCCATTGTGCTCCGGCAACGACGGCGCAGAAAAAGCCTGCCAGGGGAGAAATCGCAACTGTTAATTTTTTATTTCCACGGGTAGAATGCTCGCC G

pyrGp1 CTAAGGGAATTACGCGGTCAAGCGCCATTTGTGTCATTTTTTAAATGACAAGCGCTTGATTTGCGTCAAAAACATTTACCCCAAAGGGGCTATTTTCTCA C

pyrGp2 CCAAAGGGGCTATTTTCTCACTCCTGATTTCAATAGTGCGCTGGCGAAGAGGAGGGATAATGAAAGTTTGTGGCACAGGTCATGTTCGGGTATACTGCTT T

pyrH TCAAAAAGGAGCCGCCTGAGGGCGGCTTCTTTTTGTGCCCATCTTGTAAATTCAGCTAACCCTTGTGGGGCTGCGCTGAAAAGCGACGTACAATGTCGCT A

trxC CCGCGCGGTTCGCCATGATTTCGTACCAAAGCCTGCGACTATCATACCTATTGAATAAAACAGATTGTTGTCTGGAACAATGTCCCCGATAATATGTAAC A OxyR

tsxp1 ACGTATCCACGACATTCAACCTGTTATGCAGTAAACGTATTTCGGGACGATTTTGTGCGTCCCGCAACATCTTTCCCCGTCATTTTGTTACTCTGCTTAC A DeoR

tsxp2 CCGCCCGAATGTGTGTAAACGTGAACGCAATCGATTACGTAAATGATAGAACTGTGAAACGAAACATATTTTTGTGAGCAATGATTTTTATAATAGGCTC C CRP-cAMP, CytR
